# NeoaPred: a deep-learning framework for predicting immunogenic neoantigen based on surface and structural features of peptide–human leukocyte antigen complexes

**DOI:** 10.1093/bioinformatics/btae547

**Published:** 2024-09-14

**Authors:** Dawei Jiang, Binbin Xi, Wenchong Tan, Zixi Chen, Jinfen Wei, Meiling Hu, Xiaoyun Lu, Dong Chen, Hongmin Cai, Hongli Du

**Affiliations:** School of Biology and Biological Engineering, South China University of Technology, Guangzhou 510006, China; School of Biology and Biological Engineering, South China University of Technology, Guangzhou 510006, China; School of Biology and Biological Engineering, South China University of Technology, Guangzhou 510006, China; School of Biology and Biological Engineering, South China University of Technology, Guangzhou 510006, China; School of Biology and Biological Engineering, South China University of Technology, Guangzhou 510006, China; School of Biology and Biological Engineering, South China University of Technology, Guangzhou 510006, China; International Cooperative Laboratory of Traditional Chinese Medicine Modernization and Innovative Drug Discovery of Chinese Ministry of Education (MOE), School of Pharmacy, Jinan University, Guangzhou 510632, China; Fangrui Institute of Innovative Drugs, South China University of Technology, Guangzhou 510006, China; School of Computer Science and Engineering, South China University of Technology, Guangzhou 510006, China; School of Biology and Biological Engineering, South China University of Technology, Guangzhou 510006, China

## Abstract

**Motivation:**

Neoantigens, derived from somatic mutations in cancer cells, can elicit anti-tumor immune responses when presented to autologous T cells by human leukocyte antigen. Identifying immunogenic neoantigens is crucial for cancer immunotherapy development. However, the accuracy of current bioinformatic methods remains unsatisfactory. Surface and structural features of peptide–HLA class I (pHLA-I) complexes offer valuable insight into the immunogenicity of neoantigens.

**Results:**

We present NeoaPred, a deep-learning framework for neoantigen prediction. NeoaPred accurately constructs pHLA-I complex structures, with 82.37% of the predicted structures showing an RMSD of < 1 Å. Using these structures, NeoaPred integrates differences in surface, structural, and atom group features between the mutant peptide and its wild-type counterpart to predict a foreignness score. This foreignness score is an effective factor for neoantigen prediction, achieving an AUROC (Area Under the Receiver Operating Characteristic Curve) of 0.81 and an AUPRC (Area Under the Precision-Recall Curve) of 0.54 in the test set, outperforming existing methods.

**Availability and implementation:**

The source code is released under an Apache v2.0 license and is available at the GitHub repository (https://github.com/Dulab2020/NeoaPred).

## 1 Introduction

Somatic mutations in cancer can give rise to neoantigens that trigger an anti-tumor immune response when presented by HLA and recognized by autologous T cells. Due to their advantages of tumor specificity and immunogenicity, neoantigens are considered promising targets for immunotherapy (Wells *et al.* 2020, Xie *et al.* 2023). Currently, many neoantigen-based immunotherapeutic strategies have been developed. Neoantigen vaccines, including long-peptide vaccines, RNA vaccines, and dendritic cell vaccines, as well as adoptive cell therapy with neoantigen-reactive T cells, have shown efficacy in inducing tumor rejection (Gubin *et al.* 2014, Carreno *et al.* 2015, Sahin *et al.* 2017, Keskin *et al.* 2019, Kristensen *et al.* 2022).

**Figure 1.**
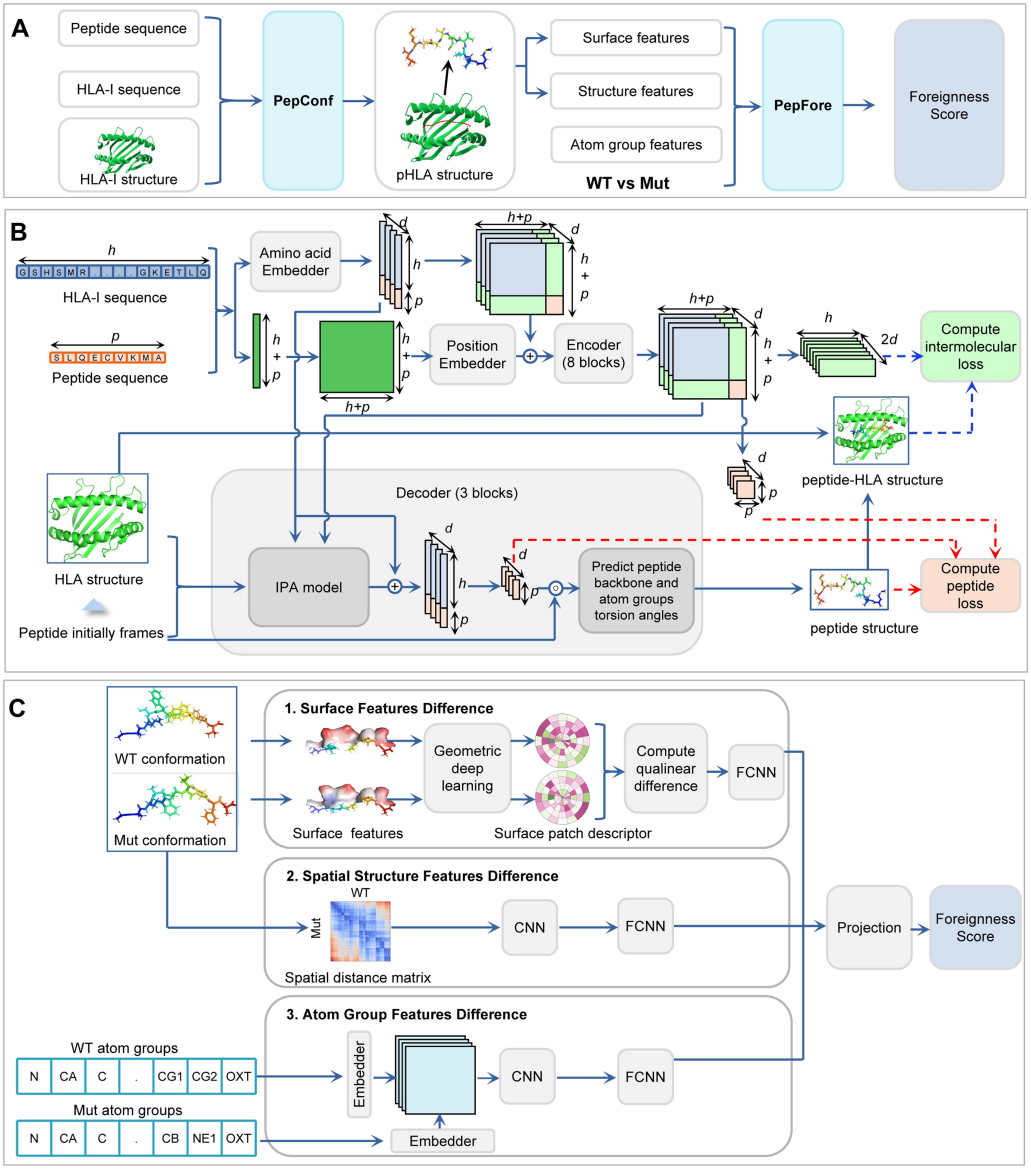
Architecture of NeoaPred. The rectangles represent different components. The arrows indicate the information flow among the components. (A) Overview of the NeoaPred workflow. PepConf: peptide conformation prediction model; PepFore: peptide foreignness score prediction model. (B) Model architecture of PepConf. *h* is the length of HLA-I sequence; *p* is the length of peptide sequence; *d* is the number of channels; dashed arrows are the algorithm workflow of *L*_pHLA_ and *L*_pep_. (C) Model architecture of PepFore. CNN, convolutional neural network; FCNN, fully connected neural network.

The effective identification of neoantigens generally relies on two key factors: the binding affinity of peptides to HLA molecules, and the foreignness of the mutant peptides. The complex formed by the binding of peptides to HLA molecules is the target of T-cell receptors, making their binding strength crucial for neoantigen recognition by T cells. Foreignness, on the other hand, refers to the “non-self” characteristic of an antigen as recognized by the host immune system. Neoantigens with a high degree of foreignness are more likely to be recognized as threats and consequently stimulate an immune response (Lang *et al.* 2022). Typically, foreignness is measured by comparing the sequence dissimilarity between the mutant peptide and the self-proteome (Richman *et al.* 2019) or by evaluating the sequence similarity between the mutant peptide and a homologous pathogenic peptide (Łuksza *et al.* 2017). Both methods focus on the sequence characteristics of peptides. However, the three-dimensional structure and physicochemical properties of molecular surfaces are also important features of peptides that should not be overlooked when calculating foreignness. In light of this, we present NeoaPred (neoantigen prediction) (Fig. 1A), a deep-learning framework designed to calculate foreignness with explicit consideration of the surface and structural features of the pHLA complex.

The molecular surface features, including geometric features and chemical features, have been successfully applied to analyze protein interactions with other biomolecules (Shulman-Peleg *et al.* 2004, Gainza *et al.* 2020, 2023). In the field of neoantigen prediction, the surface and structural features of pHLA complexes also exhibit encouraging potential (Riley *et al.* 2019, Custodio *et al.* 2023). The calculation of these features is based on the spatial structure of molecules. Nevertheless, the 3D structure of pHLA complexes is primarily solved by X-ray crystallography, nuclear magnetic resonance, or electron microscopy, which are not feasible for high-throughput analysis. Additionally, existing protein structure prediction frameworks, such as AlphaFold2, OpenFold, and RoseTAAFold (Baek *et al.* 2021, Jumper *et al.* 2021, Ahdritz *et al.* 2024), are primarily designed for single-chain proteins and struggle to predict the structures of pHLA complexes (Marzella *et al.* 2022). Recently, AlphaFold2 has been optimized and upgraded to AlphaFold3 (Abramson *et al.* 2024), introducing a diffusion-based architecture that can directly predict the structure of protein complexes with high precision. Due to the extensive use of pHLA complexes during training, AlphaFold3 has the potential to accurately predict the structures of these complexes. However, its closed-source nature and limited task quotas pose challenges for use. In this study, our framework NeoaPred incorporates a deep-learning model, PepConf (Peptide Conformation), to construct the structure of pHLA-I complexes (Fig. 1B). Based on the predicted structure, NeoaPred can generate multi-dimensional molecular features and feed them into another deep-learning model, PepFore (Peptide Foreignness) (Fig. 1C). PepFore integrates the differences in surface features, spatial structure features, and atom group features between mutant (Mut) and wild-type (WT) peptides to predict a foreignness score. This foreignness score has proved to be a highly effective factor for neoantigen prediction.

Overall, we present NeoaPred, a deep-learning framework for predicting immunogenic neoantigens. NeoaPred comprises two proof-of-concept applications: (1) PepConf for predicting the conformation of peptide binding to HLA-I and (2) PepFore for predicting the foreignness score of peptide. Our results demonstrate that NeoaPred significantly improves the accuracy of neoantigen prediction.

## 2 Materials and methods

### 2.1 Collection of data for PepConf

A total of 1018 experimental structures of MHC-I complexes were collected from the Protein Data Bank (PDB) (Berman *et al.* 2000) (Supplementary Table S1A and B). The structures were parsed using the ‘*PDB*’ function from the *Biopython* package (Cock *et al.* 2009) to extract the peptide and MHC chains. Ninety percent of the PDB data were used for training and validation to develop an initial model (Supplementary Fig. S1A), while the remaining 10% were reserved as the independent test set (Supplementary Fig. S1B). The peptide lengths of PDB data range from 7 to 14, with the majority of them being 8, 9, or 10 (Supplementary Fig. S2A). Additionally, we also collected 3000 pHLA-I ligand elution data from the Immune Epitope Database (IEDB) (Vita *et al.* 2019) (Supplementary Fig. S1B, Table S1C) to evaluate model performance. These peptides have been confirmed to bind to HLA-I by specific antibody elution assay.

Due to the requirement of HLA-I structure as input for the model, we collected 200 HLA-I allele templates from PDB and AlphafoldDB (Varadi *et al.* 2022), or obtained them through homology modeling using SWISS-MODEL (Waterhouse *et al.* 2018) (Supplementary Fig. S2B, Table S2). The cumulative frequency of these alleles exceeds 0.94, ensuring the coverage of high-frequency alleles across most populations (Supplementary Fig. S2C). To simplify the model and focus on the HLA-I binding groove domain, we only retained residues 1–180 of the HLA-I molecules. The peptide sequence was padded to a maximum length of 16 residues.

### 2.2 Self-distillation data for PepConf

Self-distillation with unlabeled data has been proven to improve the accuracy of the protein structure prediction model (Xie et al. 2020, Jumper *et al.* 2021). We applied a similar approach in training PepConf, following these steps: (1) initial model training. We used PDB data for training and validation to develop an initial model. (2) Unlabeled data prediction. Using the initial model, we predicted structures for 48 930 unlabeled pHLA-I complexes with high binding affinity (IC50 < 300 nM) from the IEDB. (3) Defining filter criteria and filtering data. We introduced the predicted local-distance difference test (pLDDT) score (Jumper *et al.* 2021) to filter the unlabeled data. As a self-estimated accuracy parameter, pLDDT shows a strong correlation with LDDT-Cα (Mariani *et al.* 2013), TM-score (Zhang and Skolnick 2004), and RMSD, with Pearson’s *r* of 0.72, 0.64, and 0.66, respectively (Supplementary Fig. S3). We removed samples with pLDDT scores <92, corresponding to LDDT-Cα < 96.35, TM-score < 0.84, and RMSD > 0.32 Å (Supplementary Fig. S3). This criterion ensured that retained samples’ predicted structures closely matched true structures. (4) Final self-distillation data. After filtering, 7860 IEDB data points were retained as the self-distillation dataset (Supplementary Table S1D). The distribution of their binding affinities and pLDDT scores is shown in Supplementary Fig. S2D and E. (5) Final model training. We trained the final model using a mixture of this self-distillation data and the PDB data.

### 2.3 Architecture of PepConf

The architecture for PepConf is illustrated in Fig. 1B. PepConf is an AlphaFold2-like framework that introduces the self-attention mechanism (Vaswani *et al.* 2017). Compared to AlphaFold2, PepConf has two unique aspects. (1) pHLA spatial distance matrix. After the embedding block, PepConf computes a two-dimensional matrix to describe the interaction between the peptide and the HLA-I molecule. This matrix is further used in encoder and decoder blocks for peptide conformation construction. (2) Loss function. We exploit intermolecular loss to enforce constraints on the spatial distance between the peptide and the HLA-I molecule. The loss function is defined in Equations (1–3):

(1) $$L=L_{\text{pep}}+L_{\text{HLA}},$$

(2) $$L_{\text{pep}}=L_{\text{FAPE}}+0.3L_{\text{dist}}+L_{\text{angle}}+L_{\text{viol}},$$

(3) $$L_{\text{pHLA}}=9.5L_{\text{pHLA-FAPE}}+0.5L_{\text{pHLA-dist}},$$

where *L* represents the total per-example loss, *L*_pep_ denotes the loss of the peptide itself, and *L*_pHLA_ represents the loss between the peptide and HLA-I molecule. *L*_pep_ is composed of four auxiliary losses, as shown in Equation (2): *L*_FAPE_ is the frame aligned point error (FAPE) loss that assesses peptide atom coordinates relative to peptide rigid groups; *L*_dist_ is the cross-entropy loss for the distribution over inter-residue distances within peptide; *L*_angle_ represents the side chain and backbone torsion angle loss; and *L*_viol_ is the structural violation loss. These auxiliary losses were previously defined in AlphaFold2 and OpenFold. *L*_pHLA_ is composed of two auxiliary losses, as shown in Equation (3): *L*_pHLA-FAPE_ is the FAPE loss that assesses peptide atom coordinates relative to HLA rigid groups; and *L*_pHLA-dist_ is the cross-entropy loss for the distribution over inter-residue distances between peptide and HLA. The purpose of the *L*_pHLA_ is to attach an individual loss to the subcomponent of the model, thereby guiding the model to accurately constrain the spatial distance between the peptide and HLA-I molecule.

### 2.4 Collection of immunogenic and non-immunogenic data for PepFore

**Figure 2.**
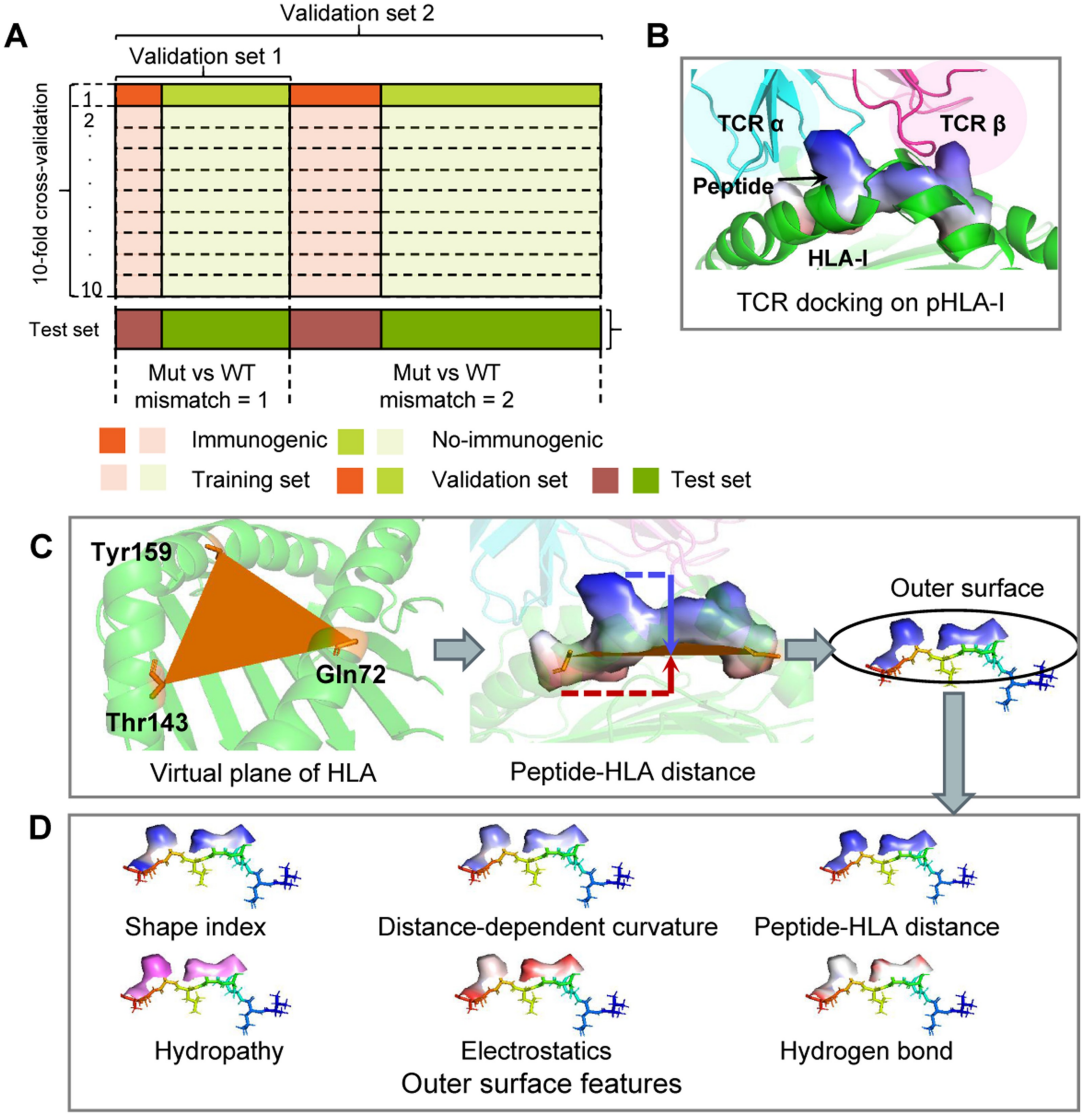
Description of the dataset for PepFore and the process of peptide surface. (A) PepFore is evaluated using standard 10-fold cross-validation with 90% of the data points (29 665). The validation set 1 is a precise data set in which WT/Mut pairs only contain one mismatch. The validation set 2 is a rough data set in which WT/Mut pairs contain one or two mismatches. Ten percent of the data points (3297) were used as the test set. (B) Schematic representation of TCR docking on pHLA-I (PDB: 1bd2). The TCR α chain and TCR β chain sit on top of the pHLA-I complex. (C) A virtual plane is determined by the three Cα atom coordinates of HLA-I (Gln72, Thr143, and Tyr159). The peptide-HLA distance is measured from the mesh on the peptide surface to the plane of the HLA-I. (D) Six outer surface features were selected as the input for the PepFore model.

We hypothesized that peptides with a high foreignness score, compared to their WT counterparts, would be less subject to self-tolerance and therefore more immunogenic. To evaluate the foreignness score, we gathered functional data that measured interferon-γ (IFN-γ) secretion upon T-cell activation by given pHLA complexes or pathogen peptide epitopes. In total, 5986 immunogenic and 26,976 non-immunogenic pHLA-I complexes data were collected from recent studies, IEDB, and IMMA2 (Tung *et al.* 2011) (Supplementary Tables S3 and S4). These peptides encompassed both cancer epitopes and pathogen epitopes, and only peptides with reported HLA-I restriction were considered. Each data point also includes a WT counterpart peptide, which was obtained by aligning the mutant or pathogenic peptide with the human genome or proteome using BLAST (Altschul *et al.* 1990). Both the blastp program (protein vs. protein) and the tblastn program (protein vs. nucleotide) were used to find the optimal match. Of the collected data points, 90% (29 665) were used for cross-validation and ablation experiments, while 10% (3297) were used as the test set (Fig. 2A, Supplementary Fig. S1C and D).

### 2.5 Precomputation of peptide surface features

The processing methods for protein surfaces have been described in references (Gainza *et al.* 2020, Gainza *et al.* 2023). In this study, we used the same methods to analyze the peptide. The peptide surfaces were initially processed into triangle meshes using the MSMS program (Sanner *et al.* 1996). Subsequently, the meshes were regularized to a resolution of 1.0 Å using pymesh (Zhou 2019). The regularized meshes were then decomposed into overlapping radial patches (Yin *et al.* 2009, Gainza *et al.* 2020). Each patch had a radius of 6.0 Å, covering a region around a central point on the peptide surface. For each patch, several different surface features were computed. (1) Shape-Index. The Shape Index describes the local curvature around each vertices of the meshes. It is defined as 2∕*π* tan^−1^((*κ*_1_ + *κ*_2_)∕(*κ*_1_ − *κ*_2_))), where *κ*_1_ and *κ*_2_ represent the principal curvatures (Gainza *et al.* 2020). (2) Distance-dependent curvature. This feature describes the relationship between any vertex *v_j_* in the patch and the center vertex *v_i_*. It is calculated as *θ(|r_j_ + n_j_* − *r_i_* − *n_i_|* − *d_ij_)|n_j_* − *n_i_|∕d_ij_*, where *θ* is a step function; *d_ij_* = | *r_j_ − r_i_* | is the distance between *v_i_* and *v*_j_; and *n_i_*, *n_j_*, *r_i_*, and *r_j_* are the normals and coordinates of *v_i_* and *v_j_*, respectively (Yin *et al.* 2009, Gainza *et al.* 2020). (3) Chemical features. Three chemical features were considered, including hydropathy, electrostatics, and hydrogen bond. These features were computed using the scripts from MaSIF (Gainza *et al.* 2020, Gainza *et al.* 2023). (4) Peptide–HLA distance. Numerous solved TCR–pHLA-I complexes have shown that αβ TCRs commonly contact the peptide–HLA-I complex in an ‘on-the-top’ binding mode (Szeto *et al.* 2020) (Fig. 2B). This conserved localization ensures that the outer surface of peptides is exposed to TCR for recognition. To quantify this exposure, we define the peptide–HLA distance as the spatial separation between the peptide’s surface and the plane of the HLA groove. This distance was computed using the method illustrated in Fig. 2C and D. Empirically, we determined that patches with a peptide–HLA distance > 4.0 Å were likely exposed to TCR. Consequently, these patches were selected to represent the outer surface of the peptide in our analysis.

### 2.6 Immunogenic peptides for feature analysis

We investigated the molecular features of six immunogenic peptides, as shown in Table 1. *KDM5C* E656K and *TENM3* A2490V are associated with ovarian cancer (Bobisse *et al.* 2018, Tanyi *et al.* 2018), *HERC1* P3278S and *OXSM* K109T with lung non-small cell carcinoma (Rizvi *et al.* 2015, Bulik-Sullivan *et al.* 2019), and *SNX24* P132L and *PGM5* H469Y with melanoma (Stronen *et al.* 2016). We predicted the conformation of these peptides using PepConf and computed their surface and structural features for comparison with their WT counterparts.

**Table 1.** Characteristics of neoepitopes and their WT counterparts.

Mutant peptide	HLA-I	Sequence^a^	Affinity (nM) (WT/Mut)^b^	RMSD (Å)^c^	TM-score^c^
*KDM5C* E656K	A0211	KMAACP[E→K]KL	40.09/251.67	0.13	0.94
*HERC1* P3278S	A1101	ASNA[P→S]SAAK	21.93/21.11	0.70	0.43
*SNX24* P132L	A0201	KLSHQ[P→L]VLL	85.09/25.78	0.18	0.91
*PGM5* H469Y	A0201	AVGS[H→Y]VYSV	91.49/29.29	0.15	0.93
*OXSM* K109T	C0304	FVS[K→T]SDIKSM	929.87/565.01	0.52	0.67
*TENM3* A2490V	A0211	GAQSWLWF[A→V]	109.77/5.66	1.23	0.41

a Peptide sequence, with the mutation in the neoepitope in brackets.

b Predicted affinity in nanomolar for peptide binding via Netmhcpan-4.1.

c Measured RMSD and TM-score between the neoepitope and WT counterpart when all common atoms of the peptides are superimposed.

### 2.7 Architecture of PepFore

The architecture for PepFore is illustrated in Fig. 1C. The WT/Mut difference information is modeled through three feature processing blocks. (1) Outer surface feature comparison block. We introduce the geometric deep-learning (Yin *et al.* 2009, Gainza *et al.* 2020) method to compute angular and radial coordinates that enable the block to map peptide surface features in a 2D Euclidean tensor. Once the mapping is performed, a convolutional neural network (CNN) is used to generate a numerical vector descriptor. Then, we use a qualinear difference function to compute the difference of descriptors between Mut and WT. The qualinear difference function is defined as

(4) $$\text{QuaLinearDifference}=w(x,y,{\left(x⊙y\right)}^{-1},|x-y|)),$$

where *x* and *y* are tensor objects for comparison, ***w*** is a trainable weight matrix, and ⊙ represents element-wise multiplication. Lastly, we use a fully connected neural network (FCNN) to extract the surface feature difference. (2) Spatial structure comparison block. We compute a spatial distance matrix between the 3D structure of WT and Mut. This matrix enables the comparison of the spatial arrangements of the atom groups, facilitating the understanding of the effects of mutations on the peptide's conformation. After that, we use CNN and FCNN to extract structural features from the matrix. (3) Atom group comparison block. We use a character embedding block to create a unique embedding for WT and Mut atom groups. Then, the embedding results (*e_w_*, *e_m_*) are modeled by an atom comparison block to capture the atomic difference. The block computes a broadcast matrix as follows:

(5) $$\text{Matrix}=\text{Concat }(B(e_{w}⊙e_{m}),|B(e_{w}-e_{m})|),$$

where ⊙ represents element-wise multiplication, and *B* represents NumPy’s broadcasting operation. Subsequently, the matrix is fed to CNN and FCNN to extract the atom group difference. At the end of the PepFore model, we apply a projection block to combine the outputs from different blocks and return a predicted foreignness score, from which we calculate the L2 loss and optimize the gradient.

### 2.8 Comparison to existing tools

Current neoantigen prediction tools can be categorized into four main approaches: (1) Affinity-based prediction. The capability of a mutant peptide to bind to MHC molecule is a fundamental requirement for T-cell recognition. We used four predictors of MHC molecule binding for comparison: MixMHCpred (Bassani-Sternberg *et al.* 2017), NetMHCpanEL, NetMHCpanBA (Reynisson *et al.* 2020), and MHCflurry (O’Donnell *et al.* 2020). These tools are machine-learning-based model or motif-based neural networks trained on pMHC data from affinity measurement experiments. The logarithm of percentile rank values (−log10%rank) is used for their results. (2) Differential Agretopicity Index (DAI)-based prediction, which compares the binding affinities between mutant and WT peptides (Ghorani *et al.* 2018). A mutant peptide with higher affinity than its WT counterpart is more likely to be an effective neoantigen (Wells *et al.* 2020). We calculated the affinity ratio based on NetMHCpanBA (NetMHCpanBA_AR) and MHCflurry (MHCflurry_AR) for comparison. (3) Immunogenic epitope prediction model. We used two predictors of immunogenic epitopes for comparison: PRIME (Schmidt *et al.* 2021) and BigMHC (Albert *et al.* 2023). PRIME uses a logistic regression model to predict the immunogenicity of epitopes. The model incorporates two key factors: The predicted HLA binding affinity and the frequency of each amino acid at positions with minimal impact on HLA binding (MIA positions). BigMHC predicts immunogenic neoepitopes using a deep neural network model that is initially trained on pMHC eluted ligand data and further fine-tuned through transfer learning on immunogenicity data. (4) Foreignness-based prediction. Two sequence-based predictors of foreignness were used for comparison: SimToIEDB (Similarity-to-IEDB) (Łuksza *et al.* 2017) and DisToSelf (Dissimilarity-to-Self-Proteome) (Richman *et al.* 2019), both available at https://github.com/immune-health/antigen.garnish. SimToIEDB evaluates the sequence similarity between neoantigen epitopes and pathogen-associated epitopes in IEDB. Higher similarity indicates an increased likelihood of cross-reactivity with preexisting T cells directed against common pathogens (Lang *et al.* 2022). DisToSelf calculates the sequence dissimilarity between neoantigen epitopes and the self-proteome. Higher dissimilarity may suggest a reduced likelihood of immune tolerance.

## 3 Results

### 3.1 Peptide conformation prediction using PepConf

**Figure 3.**
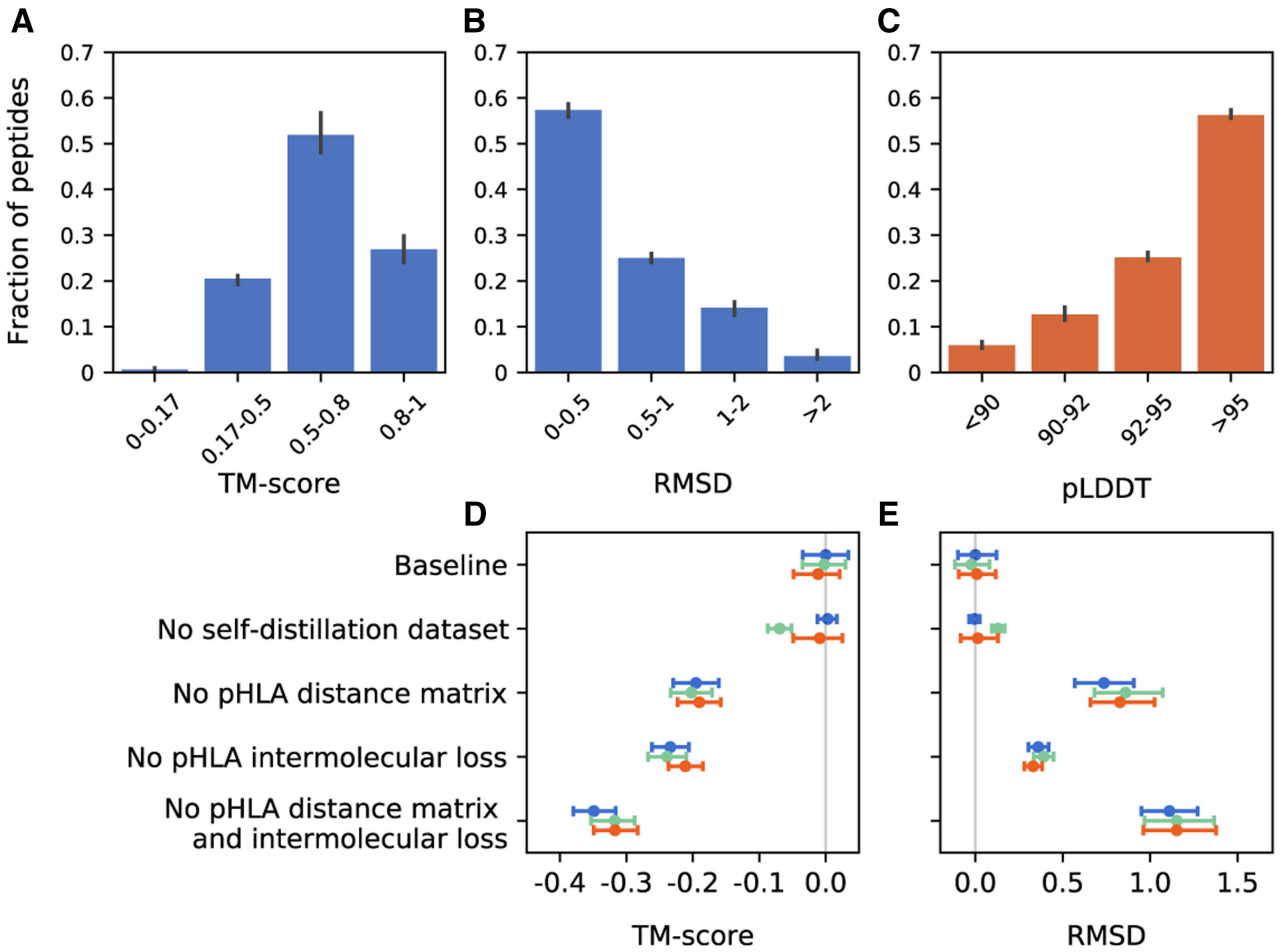
Accuracy and ablation results of PepConf. Error bars show a 95% confidence interval. (A, B) Histograms of TM-score and RMSD for PDB test data (*n* = 104). (C) Histogram of pLDDT for IEDB test data (*n* = 3000). (D, E) Ablation results of PepConf. TM-score and RMSD are selected as structure accuracy metrics. Each ablation group is replicated three times, with different colors indicating each repetition. The ablations are reported as a difference compared with the average of the three baseline seeds. Baseline: full model with self-distillation dataset training. No self-distillation dataset: full model without self-distillation dataset training. No pHLA distance matrix: we set the part of the representation matrix that describes the spatial distance between the peptide and HLA-I molecule to zero. No pHLA intermolecular loss: we remove the pHLA intermolecular loss.

To validate the accuracy of PepConf, we first generated conformations for 104 test samples collected from PDB. In this test set, 82.37% of peptides had an RMSD value of <1 Å and 78.85% had a TM-score of >0.5 (Fig. 3A and B), indicating that PepConf can produce highly accurate peptide conformations. Next, we generated conformations for 3000 unlabeled samples screened from IEDB. We evaluated the accuracy of their conformations using pLDDT, which has a strong correlation with LDDT-Cα, TM-score, and RMSD, with Pearson's correlation coefficients (*r*) of 0.72, 0.64, and 0.66, respectively (Supplementary Fig. S3). In the IEDB test set, 81.40% of peptides had a pLDDT > 92 (Fig. 3C), confirming the reliability of the prediction results.

The performance of PepConf on different lengths of peptides was also evaluated. PepConf exhibits the best performance on 8-mer and 9-mer peptides, achieving median RMSDs of 0.32 and 0.34 Å, along with median TM-scores of 0.76 and 0.73, respectively. This performance is slightly better than that for 10-mer peptides, which have a median RMSD of 0.52 Å and a median TM-score of 0.61 (Supplementary Fig. S4A). Additionally, we evaluated the predictive performance of PepConf for the HLA-A, HLA-B, and HLA-C alleles. Despite the relative scarcity of training data for HLA-C compared to HLA-A and HLA-B (Supplementary Fig. S1A), no significant differences were observed among them (Supplementary Fig. S4B). However, the absence of alleles does affect the predictive performance of PepConf. When we removed some alleles from the training set, we observed a slight decrease in the prediction accuracy for these missing alleles (Supplementary Fig. S4C). The impact of HLA-I template sources on prediction accuracy was also evaluated using 3000 IEDB ligand elution data. For HLA-I alleles sourced from AlphaFold DB, PDB, and SWISS-MODEL, there were no significant differences in predictive accuracy among them (Supplementary Fig. S4D).

Furthermore, we assessed the importance of some components designed in PepConf. The ablation study shows that the spatial distance matrix and intermolecular loss between the peptide and HLA-I molecule were crucial to enhancing PepConf's performance (Fig. 3D and E). Conversely, the impact of self-distillation data was relatively small. Adding self-distillation data to the training set did not lead to a significant decrease in evaluation loss (Supplementary Fig. S5).

Overall, NeoaPred-PepConf is able to generate highly accurate peptide conformations for subsequent analysis.

### 3.2 Performance comparison between PepConf and PANDORA

**Figure 4.**
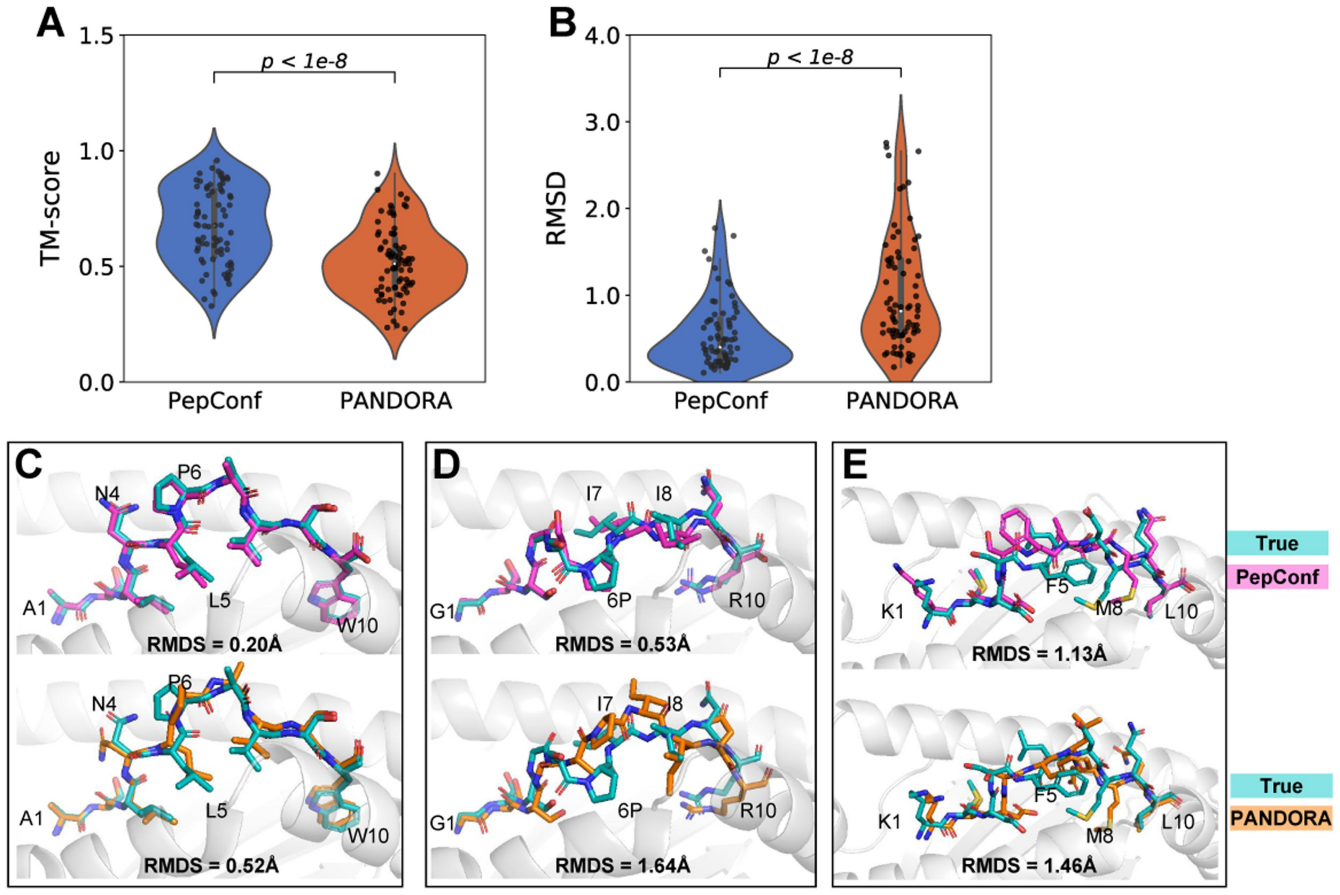
Modeling results of PepConf and PANDORA. (A, B) Comparison of peptide TM-score and RMSD between PepConf and PANDORA on the PDB test set (*n* = 104). For each data point, PANDORA generated 20 pHLA complex structures, and the modeling structure with the best molpdf score is selected as the final result. The *P*-value is computed using the Wilcoxon signed-rank test. (C, E) Comparison of the modeled structure between PepConf and PANDORA. For (C), the peptide sequence is ASLNLPAVSW, and the HLA allele is B*57:03 (PDB: 6v2p). For (D), the peptide sequence is GTSGSPIINR, and the HLA allele is A*11:01 (PDB: 5wkh). For (E), the peptide sequence is KMDSFLDMQL, and the HLA allele is A*02:01 (PDB: 3bhb).

We also compared PepConf to PANDORA, a homology modelling framework for pHLA complexes, using the PDB test set. The RMSD and TM-score of peptides obtained by the two methods are shown in Fig. 4A and B. PepConf demonstrated superior performance with a mean RMSD of 0.53 Å, significantly lower than PANDORA’s 1.01 Å. Moreover, PepConf achieves a mean TM-score of 0.67, outperforming PANDORA’s 0.52. For a more detailed comparison, we illustrated the modeling structure in Fig. 4C–E. For the peptide ASLNLPAVSW bound to HLA-B*57:03 (PDB: 6v2p), the PepConf model exhibited excellent agreement with true structure, achieving an RMSD of 0.20 Å. In contrast, PANDORA performed less favorably, mis-modeling the torsion angles of the side chain at positions 4–6. For the peptide GTSGSPIINR bound to HLA-A*11:01 (PDB: 5wkh), PepConf slightly misplaced the isoleucine residues at positions 7 and 8, with an RMSD of 0.53 Å. PANDORA only correctly modeled the N-terminus of the peptide, resulting in an RMSD of 1.64 Å. For the peptide KMDSFLDMQL bound to HLA-A*02:01 (PDB: 3bhb), both PepConf and PANDORA mis-modeled the peptide’s central bulge from positions 5 to 8.

### 3.3 Conformational characteristic of peptide binding to the HLA-I

The amino acids at the C- and N-terminus of peptides are generated by proteasomes and aminopeptidases (Guillaume *et al.* 2010, Admon 2019) and serve as primary anchors for HLA binding (Alvarez *et al.* 2019, Kotsias *et al.* 2019). This anchoring role implies that these terminal regions are in close spatial proximity to HLA molecules. Our observations reveal an ‘arch’ conformation adopted by peptides when binding to the HLA-I grooves, further supporting this concept. To demonstrate this, we examined the conformation of three pHLA-I complexes from PDB: 5hga (8-mer), 6uk2 (9-mer), and 4gfp (10-mer) (Supplementary Fig. S6A). Notably, the C- and N-terminal residues of peptides bend downward, while the middle residues bulge upward. To better understand the conformational characteristics of peptides, we measured the interaction strengths of the residues between the peptide and HLA. The strength is defined as the inverse square of the minimum atom distance. In the experimental data from the PDB, the residues at the termini exhibited much higher strengths than those in the middle (Supplementary Fig. S6B). The IEDB data predicted by PepConf also showed a similar pattern (Supplementary Fig. S6C).

This conformational characteristic indicates that the central bulge of the peptide may more easily contact with TCR. Therefore, to enhance our prediction of immunogenic neoantigens, we focus on these bulging regions by imposing restrictions on the peptide–HLA distance (see Methods).

### 3.4 Surface and structural features from WT to immunogenic peptide

Foreignness scores, calculated based on sequence difference, have been demonstrated to aid in neoantigen prediction (Łuksza *et al.* 2017, Richman *et al.* 2019, Wells *et al.* 2020). We propose that features sampled from molecular surfaces and conformation also contain valuable information for deciphering the foreignness of mutant peptides, potentially providing insights into their immunogenicity. To verify this concept, we investigated the molecular features of six immunogenic peptides and their WT counterparts (Table 1). These peptides are tumor DNA mutation-derived neoantigens and have been experimentally confirmed to stimulate T cells to elicit an anti-tumor response.

**Figure 5.**
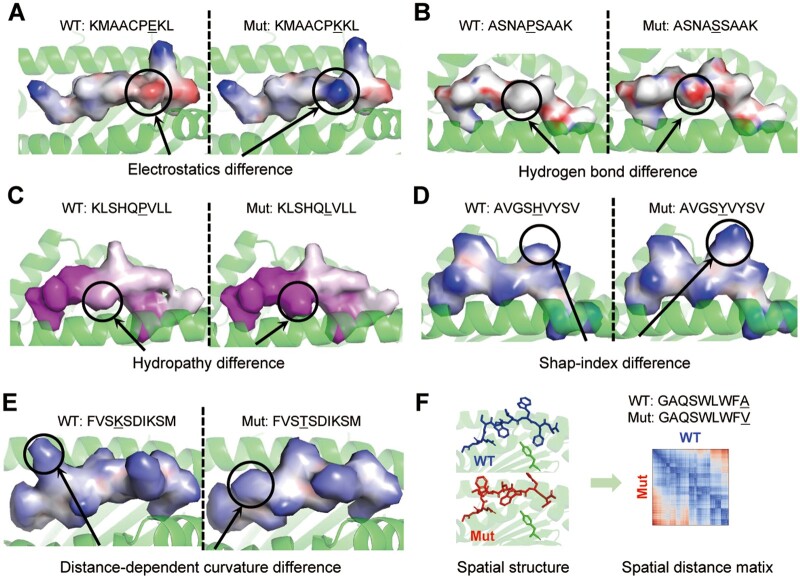
Comparison of the surface and structural features between Mut and WT counterparts. (A) Electrostatic features between *KDM5C* E656K (positive charge) and WT (negative charge). (B) Hydrogen bond features between *HERC1* P3278S (hydrogen bond donors) and WT. (C) Hydropathy features between *SNX24* P132L (hydrophobic) and WT. (D) Shape-index features between *PGM5* H469Y and WT. (E) Distance-dependent curvature features between *OXSM* K109T and WT. (F) Spatial structure and atom spatial distance matrix of *TENM3* A2490V and WT.

Differences in surface features were indeed observed in immunogenic peptides. For the epitope of *KDM5C* E656K, the mutation of negatively charged glutamic acid to positively charged lysine alters the local electrostatic property of the peptide surface (Fig. 5A). For *HERC1* P3278S, the mutation of proline to serine enhances the formation of hydrogen bonds (Fig. 5B). This can be attributed to the fact that proline has a pyrrolidine loop in its side chain, while serine has a hydroxyl group, which is considered potential donor or acceptor. For *SNX24* P132L, the mutation of proline to leucine, which has a long non-polar hydrocarbon chain, increases the hydrophobic surface area of the peptide (Fig. 5C). For *PGM5* H469Y and *OXSM* K109T, the mutations alter the geometric features of the peptide surface. Visualization of the shape-index and distance-dependent curvature revealed different protrusions on the WT and Mut peptide surfaces (Fig. 5D and E). The RMSD of these five peptides relative to their WT counterparts ranges from 0.13 to 0.70 Å (average 0.33 Å, Table 1), indicating relatively minor structural changes caused by these mutations.

In another peptide, structural features difference may be related to immunogenicity. For the epitope of *TENM3* A2490V, the C-terminal alanine to valine mutation introduces a large structural change, reflected in the diagonal asymmetry of the spatial distance matrix (Fig. 5F). With this change, the RMSD reaches ∼1.23 Å (Table 1).

Among the six cases examined, significant differences in surface or structural features were observed between the mutant peptides and their WT counterparts. These differences are likely to contribute to their foreignness. Therefore, we propose a comprehensive model, PepFore, to predict the foreignness score by considering sequence, surface, and structural features.

### 3.5 Neoantigen prediction using PepFore

**Figure 6.**
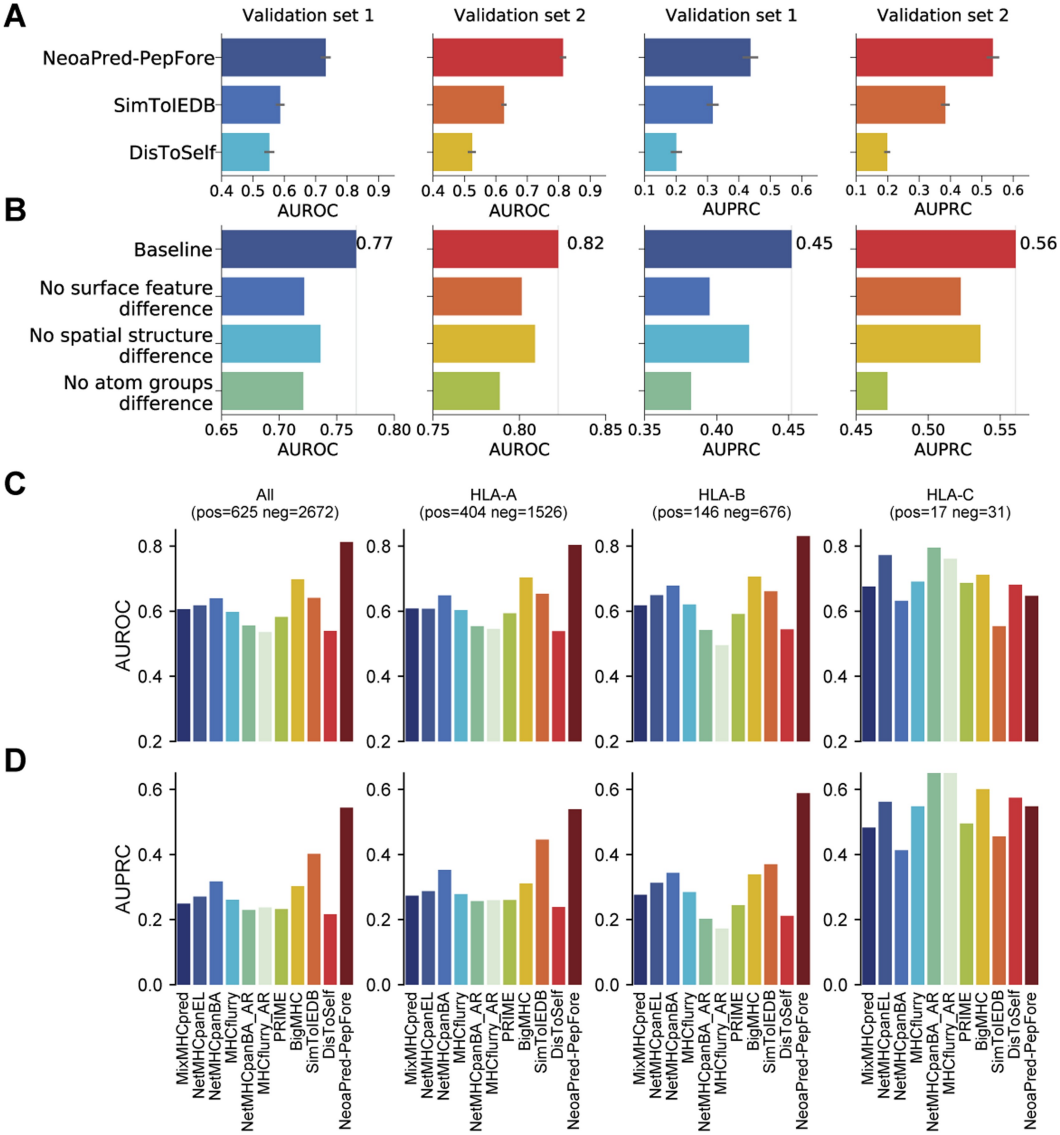
Performance comparison and ablation results of PepFore. (A) Measurement of PepFore performance by AUROC and AUPRC values of the 10-fold cross-validation in validation sets 1 and 2. The results of two other foreignness scores, SimToIEDB and DisToSelf, are also shown. (B) Sub-block ablation results for validation sets 1 and 2. Baseline: full model. No surface feature difference: we set the dissimilarity of WT/Mut surface features to zero. No spatial structure difference: we remove the WT/Mut spatial distance matrix. No atom group difference: we remove the WT/Mut atom group comparison block. (C, D) Performance comparison between PepFore and 10 common neoantigen prediction methods. We compared the performance of PepFore with 10 other methods using two metrics, AUROC and AUPRC, in the PepFore test set. The complete test set consisted of 625 positive samples and 2672 negative samples. Additionally, we evaluated the predictive performance separately for the HLA-A, HLA-B, and HLA-C alleles. The number of samples for each allele is indicated in the figure.

We trained and evaluated the PepFore model using standard 10-fold cross-validation on two validation sets with different precision levels (Fig. 2A). For comparison, we considered two additional foreignness scores based on sequence difference: SimToIEDB and DisToSelf. In validation set 1, NoeaPred performed the best, with average AUROC (Area Under the Receiver Operating Characteristic Curve) and AUPRC (Area Under the Precision-Recall Curve) scores of 0.73 and 0.43, respectively. SimToIEDB followed with 0.59 and 0.39, while DisToSelf showed lower performance with 0.55 and 0.19 (Fig. 6A; Supplementary Table S5). The results in validation set 2 were similar. Our results indicated that integrating surface and structural features can enhance prediction accuracy compared to methods that solely depend on sequence information.

However, PepFore’s predictive capacity for alleles absent from the training set is diminished. When certain alleles were completely removed from the training set, PepFore exhibited AUROC and AUPRC values of 0.62 and 0.61, respectively, for these missing alleles. In contrast, for the alleles present in the training set, PepFore achieved AUROC and AUPRC of 0.73 and 0.71, respectively (Supplementary Fig. S7A). The edit distance between WT and Mut also shows a strong correlation with PepFore's predictive capability. As the edit distance increases, the predictive sensitivity improves, while the specificity slightly decreases. Nevertheless, the overall AUROC and AUPRC are not affected by the edit distance (Supplementary Fig. S7B).

Furthermore, we assessed the influence of different feature processing blocks in PepFore. Firstly, we trained PepFore using the full model, obtaining an AUROC of 0.77 and an AUPRC of 0.45 in validation set 1. Then we removed the blocks of surface features difference, spatial structure difference, and atom group difference, which reduced the AUROC to 0.72, 0.74, and 0.72, and reduced the AUPRC to 0.40, 0.42, and 0.38, respectively (Fig. 6B). Similar ablation results were observed in validation set 2. These results demonstrated that all three feature processing blocks contributed to the performance of PepFore.

### 3.6 Performance comparison between PepFore and common methods

To further examine the effectiveness of PepFore’s foreignness score in neoantigen prediction, we compared it with several common methods, including MixMHCpred, NetMHCpan, MHCflurry, NetMHCpanBA_AR, MHCflurry_AR, PRIME, BigMHC, SimToIEDB, and DisToSelf, on a test set of 625 immunogenic and 2,672 non-immunogenic peptides. The results, illustrated in Fig. 6C and D, indicate that PepFore achieved the highest performance with an AUROC of 0.81 and an AUPRC of 0.54. The best prior method was BigMHC, achieving an AUROC of 0.70 and an AUPRC of 0.30, while SimToIEDB followed closely with an AUROC of 0.64 and an AUPRC of 0.40. We further assessed the prediction accuracy across different HLA alleles. Our analysis revealed that PepFore demonstrated superior accuracy in predicting neoantigens for HLA-A and HLA-B alleles. However, its performance was significantly lower for HLA-C alleles (Fig. 6C and D, Supplementary Table S6), potentially due to the scarcity of HLA-C instances within the training dataset (Supplementary Fig. S1C).

Overall, by considering both surface and structural features, PepFore offers a more comprehensive assessment of peptide foreignness, enhancing the accuracy of neoantigen identification.

## 4 Discussion

The successful prediction of neoantigens is dependent on understanding the parameters that govern immunogenicity (Wells *et al.* 2020). Previous methods have been mostly restricted to predicting the binding of pHLA complexes or analyzing the sequence of mutant peptides, which is necessary but not sufficient (Bassani-Sternberg *et al.* 2017, Łuksza *et al.* 2017, Richman *et al.* 2019, O’Donnell *et al.* 2020, Reynisson *et al.* 2020, Kim *et al.* 2023). Recently, the importance of structural features has been recognized (Custodio *et al.* 2023). However, the structures of pHLA complexes are mainly solved through electron crystallography methods, which are not suitable for high-throughput prediction of neoantigens. Our general framework (NeoaPred) provides a new method that may overcome these barriers and enable a more complete understanding of neoantigens. The NeoaPred-PepConf model is specifically designed for predicting the conformation of peptides binding to HLA-I. Despite its early-stage development, PepConf can be useful in understanding the structural relationship between peptides and HLA-I molecules. The NeoaPred-PepFore model showcases the potential of surface and structural features for immunogenic neoantigen identification. By comparing these features between neoantigen and its WT counterpart, we can achieve a rationalized foreignness score. We anticipate that NeoaPred will be especially important for neoantigen prediction.

However, there are several limitations to this study. Firstly, predicting the conformation of peptide binding to HLA-II remains an unsolved challenge. HLA-II-restricted neoantigens have been proven crucial for some antitumor responses (Kreiter *et al.* 2015, Khodadoust *et al.* 2017). However, the performance of relevant bioinformatic tools remains poor due to the paucity of training data (Boegel *et al.* 2019). In the future, a transfer learning network-based approach (Lin *et al.* 2023) may be suitable to address this task. Secondly, the training data of the PepConf model are limited and unevenly distributed across different alleles, with a notable scarcity of HLA-C data. Despite efforts to mitigate this issue by introducing self-distillation data, the resultant enhancement in model performance was modest. Similarly, the PepFore model encounters this data limitation, leading to significantly lower predictive performance for HLA-C compared to HLA-A and HLA-B. Consequently, predictions for HLA-C warrant particular caution in interpretation. Another limitation is that the foreignness score is not the only factor affecting the efficacy of neoantigens (Wells *et al.* 2020). Other factors, such as antigen processing and antigen presentation, also need to be considered when identifying neoantigens.

In conclusion, we present a novel method to predict the conformation of the pHLA complex and decipher the neoantigen foreignness by comparing the representation of peptide surfaces and structures, along with atom groups. Compared to previous studies, our framework provides a unique perspective on the immunogenicity of neoantigens and proposes an important method for enhancing computational neoantigen prediction.

## Supplementary Material

btae547_Supplementary_Data
