# Supplementary material for: NeoaPred: a deep-learning framework for predicting immunogenic neoantigen based on surface and structural features of peptide–human leukocyte antigen complexes
Source: Bioinformatics. 2024 Sep 14;40(9):btae547. doi: 10.1093/bioinformatics/btae547 (PMC11419954; doi:10.1093/bioinformatics/btae547)
Supplement: btae547_Supplementary_Data [file btae547_supplementary_data.zip › Supplementary file1.doc]

# Supplementary Figures S1 to S7:

Figure S1. Description of the HLA allele frequencies in the datasets. The pie charts illustrate the proportions of different types of HLA-I genes in various datasets. The bar charts illustrate the alleles with a frequency > 0.005 in the datasets. (A) Data for PepConf model training. A total of 914 PDB samples (90% of all PDB data) and 7,860 self-distillation samples were used for training. Non-classical HLA-I includes HLA-E, HLA-G, and HLA-F. Non-human MHC-I samples are derived from *Mus musculus*, *Sus scrofa*, and other species. (B) Data for PepConf model testing. A total of 104 PDB samples (10% of all PDB data) and 3,000 IEDB ligand elution samples were used for testing. (C) Data for model training. 90% of PepFore data is used for model training, which includes 5,361 positive samples and 24,304 negative samples. (D) Data for PepFore model testing. 10% of PepFore data is used for model training, which includes 625 positive samples and 2,672 negative samples.

Figure S2. Description of the datasets and HLA-I templates. (A) Distribution of peptide lengths in the datasets. (B) Sources of the 200 HLA-I templates. (C) Overall frequencies of the selected 200 HLA templates across different populations. The lowest overall frequency (0.94) is observed in the HLA-B gene among the South Asian Indian population. (D) Distribution of binding affinities within the self-distillation dataset for pHLA complexes, with affinity measured in terms of IC50 (nM). (E) Distribution of pLDDT scores for pHLA complexes in the self-distillation dataset.

Figure S3. Correlation between pLDDT and prediction accuracy metrics **(LDDT-Cα, TM-score, and RMSD).** The shaded region of the linear fit represents a 95% confidence interval estimated from 1000 bootstrap samples. Least-squares linear fit LDDT-Cα = 2.08×pLDDT−95.01; TM-score = 0.04×pLDDT−2.84; RMSD = −0.13×pLDDT＋12.28

Figure S4. Performance testing of PepConf. (**A-B**) Prediction accuracy of peptides with different lengths and HLA-I alleles. TM-scores and RMSD are estimated in the PDB test set, and pLDDT scores are estimated in the IEDB test set. The values labeled in the chart represent the median for each group. P-values are computed using the Mann-Whitney U rank test. (**C**) Capability of PepConf to predict HLA-I alleles absent from the training set. Ten alleles were removed from the PDB training set, and then the PepConf model was trained from scratch. The resulting model was used to evaluate its performance on alleles present or absent in the training set. P-values are computed using the Mann-Whitney U rank test. (**D**) Impact of template source on PepConf performance. We compared the pLDDT values of the HLA-I allele derived from AlphaFold DB, PDB, and SwissModel using 3000 IEDB ligand elution data points. Using the Mann-Whitney U rank test, no significant differences were observed among the three groups.

Figure S5. Assessing the influence of self-distillation data on the PepConf model. We incrementally mixed self-distillation data into the PDB training set, monitoring the resultant fluctuations in evaluation loss to gauge the impact of this self-distillation data on the model's learning process.

Figure S6. Conformational characteristics of peptide binding to the HLA. (**A**) Comparisons are presented between the predicted (in red) and actual (in blue) conformations for three peptides bound to HLA. The peptide sequences are RFPLTFGW, KQWLVWLLL, and ELAAIGILTV, respectively, from the PDB entries 5hga (left), 6uk2 (middle), and 4gfp (right). (**B-C**) Interaction strengths of the residues between the peptide and HLA. Interaction strength is defined as the inverse square of the minimum atom distance. Residue positions on the peptide are denoted by P*x*, with P1 corresponding to the N-terminus. The actual interaction data from the PDB is shown in blue (**B**), while the predicted interaction data derived from IEDB ligand elution is shown in red (**C**).

Figure S7. Performance testing of PepFore. (A) Capability of PepFore to predict HLA-I alleles absent from the training set. Nine alleles were removed from the PepFore training set, and then the PepFore model was trained from scratch. The resulting model was used to assess its performance on the alleles present or absent in the training set. (B) The prediction accuracy of peptides with different edit distances in the PepFore test set. For the wild-type peptide X and mutant peptide Y, the amino acid substitution is *x* to *y*, and the edit distance was computed as *b_x,x_* - *b_x,y_*, where *b_a,b_* stands for the BLOSUM62 entry for amino acids *a* and *b*. Edit distance shows a Pearson’s correlation coefficient (*r*) of 0.72 with sensitivity and -0.84 with specificity.
